# Effects of *Saccharomyces cerevisiae* cell wall addition on feed digestibility, fecal fermentation and microbiota and immunological parameters in adult cats

**DOI:** 10.1186/s12917-021-03049-8

**Published:** 2021-11-16

**Authors:** Laura Fantucci de Oliveira Matheus, Larissa Wunsche Risolia, Mariane Ceschin Ernandes, Johnny Maciel de Souza, Patrícia Massae Oba, Thiago Henrique Annibale Vendramini, Vivian Pedrinelli, Lucas Ben Fiuza Henríquez, Cristina de Oliveira Massoco, Cristiana Fonseca Ferreira Pontieri, Marcio Antonio Brunetto

**Affiliations:** 1 School of Veterinary Medicine and Animal Science, University of São Paulo, 87, Prof. Orlando Marques de Paiva Ave, São Paulo, São Paulo 05508270 Brazil; 2 Department of Animal Sciences, University of Illinois at Urbana-Champaign, 120, 7 W Gregory Dr, Urbana, IL 61801 USA; 3 Grandfood Industria e Comercio LTDA (Premier Pet, Luiz Augusto de Oliveira Hwy, km 204, Dourado, São Paulo 13590-000 Brazil

**Keywords:** Biogenic amines, Fecal real-time PCR, Feline, Fermentation, Isovaleric acid

## Abstract

**Background:**

This study aimed to evaluate the effects of increasing dosages of a commercial product composed by *Saccharomyces cerevisiae* yeast (YAM), with active metabolites, which are beta glucans, nucleotides, organic acids, polyphenols, amino acids, vitamins and minerals (Original XPC^tm^, Diamond V, IOWA, USA) added to a commercially available dry cat food. Apparent digestibility of dietary nutrients, fecal microbiota, fecal fermentation products and immunological parameters were evaluated. Twenty-seven healthy cats of mixed sexes, with a mean body weight of 4.19 ± 0.83 kg and a mean age of 9.44 ± 5.35 years were distributed by age in an unbalanced randomized block design, consisting of three experimental treatments: CD (control diet), YAM 0.3 (control diet with 0.3% yeast with active metabolites) and YAM 0.6 (control diet with 0.6% yeast with active metabolites).

**Results:**

The inclusion of the additive elevated the apparent digestibility of crude fiber (*p* = 0.013) and ash (*p* < 0.001) without interfering feed consumption, fecal production and fecal characteristics. Regarding fermentation products present in the feces, prebiotic inclusion increased lactic acid concentration (*p* = 0.004) while reducing isovaleric acid (*p* = 0.014), only in the treatment YAM 0.3. No differences were noticed on biogenic amines (BA), fecal pH, ammonia concentration, total and individuals short-chain fatty acids (SCFA) and total and individuals branched-chain fatty acids (BCFA) (except isovaleric acid in YAM 0.3). As regards to fecal microbiota, prebiotic inclusion has resulted in the reduction of *Clostridium perfringens* (*p* = 0.023). No differences were found in the immunological parameters evaluated.

**Conclusion:**

It can be concluded that the additive, at the levels of inclusion assessed shows prebiotic potential and it has effects on fecal fermentation products and microbiota without interfering on crude protein and dry matter digestibility. More studies evaluating grater inclusion levels of the prebiotic are necessary to determine optimal concentration.

## Background

The intestinal microbiota provides the host various functions regarding physiology, especially related to metabolic and immune homeostasis [1]. Several factors can affect the intestinal microbiota, but the diet plays this role predominantly [2, 3]. It is known that dysbiosis may be associated with some disorders, such as colorectal cancer, obesity, insulin resistance, type 2 diabetes mellitus, allergic diseases, among others [4, 5] and the use of nutraceuticals can prevent it through gut microbiota modulation [6].

Nutraceuticals are substances that have the ability to improve animal health and have been increasingly studied in pet nutrition [7, 8]. An example of nutraceutical are prebiotics that can be defined as a non-digestible food ingredient that beneficially affects the host by selectively stimulating growth and/or activity of a limited number of bacteria in the colon [1, 6, 9–11]. In this context of food promoting health, prebiotics have been the subject of numerous scientific studies and there are publications which have demonstrated their therapeutic effectiveness on both systemic and gastrointestinal tract [11–16].

The *Saccharomyces cerevisiae* cell wall is a prebiotic composed of two fractions: one formed by beta-glucans and chitin and the other consisting of mannoproteins such as mannan oligosaccharides (MOS) [17]. When this yeast is dehydrated it becomes a commercial product called: Original XPC^tm^ (Diamond V., Iowa, USA), which is intended for feeding several species of domestic animals, such as cats. The recommendation of this product for cats is 2 kg/ton. This yeast with active metabolites (YAM) has a final composition of beta glucans, nucleotides, organic acids, polyphenols, amino acids, vitamins and minerals.

Considering the potential benefits of modulating intestinal microbiota and the likely prebiotic action of YAM, the objective of this experiment was to evaluate the effect of supplementing increasing levels of YAM on fecal microbiota, fecal fermentation products, digestibility, and immunological parameters of healthy adult cats.

## Results

During the study, two animals of CD and one of YAM 0.3 were excluded for not ingesting an adequate amount of food. At the end of the study, the number of animals per group was: eight of CD, nine of YAM 0.3 and ten of YAM 0.6.

Feed intake was adequate, and the animals have ingested the amount of feed proposed by NRC [18] to achieve the maintenance energy requirement. All cats maintained constant body weight (BW) during the experiment (4.29 ± 0.89 kg). The mean BW of the CD was 3.9 ± 0.89 kg; YAM 0.3 was 3.97 ± 0.54 kg and YAM 0.6 was 4.52 ± 0.90 kg. The animals did not present a significant difference in BW among treatments.

The results of the nutrient intake (g/day), fecal production (g/dry matter/day), fecal score and apparent nutrient digestibility (%) are described in Table 1. There was no difference in the average daily intake of dry matter (DM), organic matter (OM) or nutrients by the animals. Fecal production and fecal scores also did not differ. The inclusion of the prebiotic did not influence the apparent nutrient digestibility of DM, OM, crude protein (CP), acid-hydrolyzed fat (AHF), or nitrogen-free extract (NFE). For crude fiber (CF), higher digestibility was observed in the yeast-treated groups than in the CD (*p* = 0.013), similar results were observed for ash, meantime YAM 0.6 superior to YAM 0.3 (*p* < 0.001). In relation to gross energy (GE) (*p* = 0.033) from the diets, the CD was superior to the supplemented groups.

**Table 1:** Nutrient intake, fecal production, fecal score and apparent nutrient digestibility in cats fed with increasing levels of YAM

	Diets^†^						
Item	CD	SEM^‡^	YAM 0.3	SEM^‡^	YAM 0.6	SEM^‡^	*p*-value
Intake, g/day							
Dry matter	60.33	6.65	61.07	4.53	55.89	3.43	0.727
Organic matter	60.09	7.08	62.82	4.59	57.20	3.51	0.732
Crude protein	20.81	2.45	20.24	1.48	18.58	1.14	0.617
Acid-hydrolyzed fat	8.53	1.07	10.77	0.78	8.97	0.55	0.160
Crude fiber	1.13	0.14	1.48	0.10	1.41	0.08	0.109
Ash	3.67	0.46	4.67	0.34	4.55	0.24	0.187
Nitrogen-free extract	25.20	2.97	24.56	1.79	22.90	1.40	0.707
Fecal production							
Fecal production, g/DM/d	7.22	1.01	9.10	0.74	7.87	0.44	0.808
Fecal score	4.41	0.24	4.67	0.15	4.55	0.24	0.628
Apparent nutrient digestibility, %							
Dry matter	84.04	1.16	84.92	0.94	85.78	0.68	0.394
Organic matter	87.38	0.93	87.82	0.95	87.82	0.71	0.677
Crude protein	87.42	1.22	88.35	1.25	87.60	0.99	0.631
Acid-hydrolyzed fat	93.99	1.63	95.21	0.89	95.21	0.68	0.354
Crude fiber	14.60^a^	3.56	27.11^b^	3.85	33.79^b^	4.25	0.013
Ash	43.28^a^	2.85	50.10^b^	1.12	53.55^c^	1.09	< 0.001
Nitrogen-free extract	89.62	1.01	88.58	0.91	89.80	0.78	0.597
Gross energy, kcal/g	3.99^a^	0.05	3.82^ab^	0.05	3.71^b^	0.04	0.033

^a,b,c^ Values in the same row with different superscripts differ significantly

^†^Diets: CD (control diet); YAM 0.3 (control diet containing 0.3% yeast with active metabolites); YAM 0.6 (control diet containing 0.6% yeast with active metabolites)

^‡^*SEM* standard error of the mean

There was no difference in fecal pH or concentration of ammonia, acetic, propionic, or butyric acid, total SCFA, valeric, isobutyric acid or total BCFA (Table 2). Lactic acid concentration (*p* = 0.004) was lower in the CD and YAM 3.0 had a lower concentration of the isovaleric acid comparing to other treatments (*p* = 0.014) (Table 2).

**Table 2:** Fecal pH, fecal concentration of lactic acid, ammonia, short-chain fatty acids, and branched-chain fatty acids of cats fed with increasing levels of YAM

	Diets^c^						
Item	CD	SEM^d^	YAM 0.3	SEM^d^	YAM 0.6	SEM^d^	*p*-value
pH	6.00	0.14	5.94	0.12	6.08	0.11	0.756
Lactic acid, μmol/kg of DM	1.36^a^	0.34	3.42^b^	0.84	4.24^b^	1.01	0.004
Ammonia, μmol/kg of DM	140.26	21.87	133.75	10.85	163.33	12.76	0.286
Short-chain fatty acids, μmol/kg of DM							
Acetic acid	66.82	11.49	92.92	12.80	66.59	10.15	0.216
Propionic acid	32.47	6.19	27.08	4.37	30.38	5.23	0.782
Butyric acid	24.50	7.41	40.29	12.30	24.84	5.69	0.545
Total short-chain fatty acids	123.79	22.26	160.29	21.05	121.81	14.57	0.329
Branched-chain fatty acids, μmol/kg of DM							
Valeric acid	9.86	2.67	14.40	2.70	12.63	2.79	0.514
Isobutyric acid	2.31	0.32	2.30	0.41	2.71	0.34	0.694
Isovaleric acid	2.39^b^	0.50	1.85^a^	0.25	2.98^b^	0.47	0.014
Total branched-chain fatty acids	15.10	3.35	17.06	2.99	16.50	3.08	0.673

^a–b^ Values in the same row with different superscripts differ significantly

^c^Diets: CD (control diet); YAM 0.3 (control diet containing 0.3% yeast with active metabolites); YAM 0.6 (control diet containing 0.6% yeast with active metabolites)

^d^*SEM* standard error of the mean

There was no difference between groups in the fecal concentration of cadaverine, spermidine, histamine, putrescine, tyramine or total BA (Table 3). The BA: agmatine, phenylethylamine, serotonin, and tryptamine were analyzed, but these compounds were not found in the feces of any group.

**Table 3:** Biogenic amines concentrations (μmol/kg of DM) in the feces of cats fed with increasing levels of YAM

	Diets^a^						
Item	CD	SEM^b^	YAM 0.3	SEM^b^	YAM 0.6	SEM^b^	*p*-value
Cadaverine	10.47	1.54	9.14	1.20	11.99	2.09	0.481
Spermidine	0.45	0.10	0.32	0.05	0.55	0.08	0.145
Histamine	1.57	0.45	1.24	0.33	1.25	0.33	0.711
Putrescine	4.75	1.13	4.72	0.73	6.07	1.47	0.603
Tyramine	3.81	0.82	4.97	0.89	5.70	1.18	0.464
Total biogenic amines	21.03	2.89	20.41	2.63	25.56	4.48	0.519

^a^Diets: CD (control diet); YAM 0.3 (control diet containing 0.3% yeast with active metabolites); YAM 0.6 (control diet containing 0.6% yeast with active metabolites)

^b^*SEM* standard error of the mean

There was no difference in the abundance of the bacterial genera *Bifidobacterium* or *Lactobacillus* or in the combined abundance of *E. coli*, *H. alvei* and *Shigella spp.* (Table 4). The species *C. perfringens* (*p* = 0.023) showed a reduction in abundance with yeast inclusion (Table 4).

**Table 4:** Quantification of fecal bacteria by quantitative real-time PCR (log10 cfu/g of faecal DM) in cats fed with increasing levels of YAM

	Diets^c^						
Item	CD	SEM^d^	YAM 0.3	SEM^d^	YAM 0.6	SEM^d^	*p*-value
*Bifidobacterium* spp.	1.00	–	1.62	2.33	0.89	1.60	0.883
*Lactobacillus* spp.	1.00	–	0.62	0.98	0.52	1.02	0.376
*Clostridium perfringens*	1.00^b^	–	0.10^a^	0.13	0.10^a^	0.06	0.023
*E. coli* – *Hafnia alvei* – *Shigella* spp.	1.00	–	0.70	1.09	0.40	1.01	0.420

^a–b^ Values in the same row with different superscripts differ significantly

^c^Diets: CD (control diet); YAM 0.3 (control diet containing 0.3% yeast with active metabolites); YAM 0.6 (control diet containing 0.6% yeast with active metabolites)

^d^*SEM* standard error of the mean

There was no difference between groups in CD4+ or CD8+ lymphocytes, CD4+/CD8+ ratio, basal or induced oxidative bursts, phagocytosis index or the proliferative response of lymphocytes to the mitogens concanavalin A (ConA) and phytohemagglutinin (PHA) (Table 5).

**Table 5:** Lymphocyte immunophenotyping, phagocytosis test, oxidative burst and lymphoproliferation test of cats fed with increasing levels of YAM

			Diets^a^				
Item	CD	SEM^b^	YAM 0.3	SEM^b^	YAM 0.6	SEM^b^	*p*-value
CD4^+^ (T helper cells), %	24.76	2.56	28.85	2.60	26.44	3.12	0.261
CD8^+^ (cytotoxic T cells), %	11.34	1.99	10.67	1.68	11.58	1.40	0.931
CD4^+^/CD8^+^ ratio	2.67	0.44	3.27	0.55	2.30	0.30	0.260
Basal oxidative burst^c^	255.63	18.26	211.00	27.62	256.29	35.92	0.372
Induced oxidative burst^c^	1686.38	225.72	1754.00	272.30	1597.63	418.82	0.947
Phagocytosis index^c^	253.67	41.51	186.44	69.85	245.55	73.11	0.733
Concanavalin A^c^	234.88	16.02	228.36	8.81	236.71	14.00	0.917
Phytohemagglutinin^c^	660.75	21.64	608.11	36.82	583.50	36.03	0.264

^a^CD (control diet); YAM 0.3 (control diet containing 0.3% yeast with active metabolites); YAM 0.6 (control diet containing 0.6% yeast with active metabolites)

^b^SEM: standard error of the mean

^c^Values was expressed as arbitrary units of fluorescence

## Discussion

In this study the effect of increasing levels (0, 0.3 and 0.6%) of a commercial *Saccharomyces cerevisiae* cell wall product on consumption, apparent digestibility of nutrients, fecal characteristics, fermentation products, fecal BA, fecal microbiota and immunological parameters of cats were evaluated.

In previous studies of yeast cell wall (YCW) as a feed additive for dogs and cats, no difference in DM intake was observed between treatments [15, 19–22]. In relation to fecal production the same lack of difference was observed in dogs with the inclusion of the prebiotic MOS, which is also present in YAM composition [11, 12, 23]. Theodoro et al. [22] observed that the MOS fraction from YCW can modulate dogs intestinal microbiota.

The high CF digestibility of the diets containing YAM need to be considered with caution. Probably these may have occurred by calculation errors, as all diets had low fiber concentrations and, consequently small differences between fiber digestibility in basal and test diets could result in calculation errors [24]. Besides, de-Oliveira et al. [25] hypothesized that some fiber compounds can become more soluble because of microbial degradation and will not be measured in the CF analysis. For this reason, a smaller content of CF may have been identified in stool which was interpreted as a higher digestibility.

Regarding ash digestibility, a previous study evaluating the effects of dietary supplementation in cats with spray dried *Saccharomyces cerevisiae* cell wall verified an increase in this parameter [15], which corroborates our study. This effect may be caused due to an increase in bacterial content and their fermentation metabolites. For example, some bacteria can produce phytase enzyme, which can release minerals attached to phytate increasing their availability. Another example is the induction of the bioactive peptides production by some bacterial groups which also may induce greater availability of minerals [26].

In relation to GE, according to NRC [18] it is defined as the total chemical energy arising from complete combustion of a food in a calorimeter bomb. Different nutrients have different ranges of GE reported and among all nutrients the carbohydrates (including fibers) have the smaller GE content [18]. In this study the diets containing YAM addition had smaller GE than the CD which was expected since they have greater fiber content.

With the addition of YAM, it would be expected a reduction on fecal pH caused by gut microbiota fermentation, as it was observed by Middelbos et al. [19] using YCW in dogs’ diet. On the other hand, Lin et al. [21] and Theodoro et al. [22] did not observed fecal pH alteration with the addition of YCW for dogs and Santos et al. [15] also did not observed this alteration for cats. The authors believe that the level of YCW inclusion may not have been sufficient in these dosages to cause an alteration on this parameter, which can also have happened in our study.

The short chain fatty acids are the main end-products of the bacterial fermentation in the mammal’s colon, and they are considered as indicators of nutritional evaluation of ingredients rich in carbohydrates used in pet-food [27]. The main SCFA are rapidly absorbed and then metabolized by the gut epithelium, liver and muscle, and have a trophic effect on the intestinal epithelium, maintaining the mucosal defense barrier against pathogens organisms [28].

Alterations in SCFA may influence on fecal pH [29], and as for fecal pH, no differences were found on total SCFA or acetic, propionic and butyric acids. Considering SCFA, our results corroborate those presented by Santos et al. [15] that did not found difference between treated and untreated cats. However, other authors found an increase in SCFAs (acetate and total SCFA) when dogs were fed a beef-based diet with 1.4% YCW extract [30] or when cats consumed a combination of fructooligosaccharides (FOS) and galactooligosaccharides (GOS) or GOS and *Bifidobacterium pseudocatenulatum* as 0.5% of their diet [14]. Therefore, a higher inclusion of YAM or a combination of probiotics and prebiotics may be able to increase SCFAs. However, more studies are necessary to support this hypothesis.

Non digested nitrogenous compounds can be metabolized in the gut and produce putrefactive catabolites such as ammonia, BA and phenols [31]. As regards to ammonia it can contribute to colon carcinogenesis and also reduction in the height of intestinal villi [31, 32]. In the present study, the fecal concentration of ammonia was not affected by the inclusion of the prebiotic. Previous studies corroborate this finding, as the dietary inclusion of FOS and MOS did not affect the ammonia concentration in dog’s feces [11, 12, 23]. Theodoro et al. [22] also find no difference in the fecal ammonia production of dogs after eating diets with the addition of YCW. Regarding the gut fermentation profile, bacteria use N (specially ammonia) to synthesis of bacterial mass [33]. Carbohydrates (as prebiotics) can serve as energy source to fermentation and bacterial growth [6]. This process results in the decrease of luminal concentration of nitrogenous compounds and increase in fecal N (comprised by bacterial mass) concentration [34]. Higher doses of YAM may be required to imply on decreases in the concentrations of BCFA and BA measured in feces, considering that those compounds are formed by nitrogen compounds fermentation.

In contradiction to the abovementioned regarding BA, there are other studies in the literature indicating its increase with prebiotic addition [15]. According to the authors, as the gut microbiota of cats is mainly comprehended by proteolytic bacteria and they cause substrate modulation. Moreover, Santos et al. [15] argument that *saccharolytic* bacteria growth may not be sufficient to reduce proteolytic microbes through competition. Considering these different effects in BA concentration found in the literature caused by prebiotics addition it is important that more studies are developed in this regard. Despite the effect expected, the concentration of YAM found in this study was not sufficient to cause any impact in fecal BA in cats.

This study could only identify as a result of gut fermentation indicating prebiotic effect the increase in lactic acid production and the reduction of isovaleric acid with the inclusion of the additive, probably because of the low inclusion levels. Regarding isovaleric acid, the addition of 0.3% YAM decreased this BCFA showing that it could be altered with less substrate than the other acids. This reduction in isovaleric acid, which is a catabolic of proteolytic activity, was accompanied by a reduction in *C. perfringens*, which supports a theory of proteolytic bacterial modulation [35]. The increase in this BCFA with the highest supplementation of the prebiotic needs further investigation.

As regards to acid lactic, according to Gannan et al. [36] the mannoproteins found in YCW can be used to enhance lactic acid bacteria intestinal populations and control pathogens. It has also been observed in this study by the increase in lactic acid with the addition of YAM in both concentrations, 0.3 and 0.6%, despite the potentially low concentration levels of inclusion. On the whole, these results are suggestive of a higher proliferation of lactic acid bacteria, which have been indicated as the main probiotic genera for healthy intestine of mammals [37].

Regarding fecal microbiota, this study has only evaluated a few bacterial taxa and the inclusion of YAM for cats resulted in the reduction of *C. perfringens*. The modulation of *C. perfringens* in the present study corroborates the results found by Santos et al. [15] in cats supplemented with increasing levels of YCW. According to Vernazza et al. [38] the prebiotic effect of YCW is mainly caused by the ability of MOS to attach to type A fimbriae of *Salmonellae* and *Clostridiae* avoiding the adhesion to enterocytes and reducing their concentration in gut microbiota, which can explain the reduction of *C. perfringens* in our study. Furthermore, the inclusion of YAM may have had other effects on gut microbiota that were not evaluated in this study, improving the multiplication of lactic acid producing bacteria and their adhesion to the mucosa.

The inclusion of YAM in cat’s diet did not altered any of the immunological parameter analyzed in this study, which is not in accordance to the literature [19–22, 31, 39]. However, this effect may not have been found in the present study owing to difficulties in establishing optimal doses for the prebiotic effect.

## Conclusion

Supplementation of *S. cerevisiae* at concentrations of 0.3 and 0.6% in cat’s diet can impact positively the gut microbiota by reducing *C. perfringens* concentrations. As consequence of this modulation the concentration of lactic acid, a beneficial metabolite is increased. All these positive effects are acquired without interfering the digestibility of DM and CP, indicating that nutrient harnessing of the diet is preserved. The effects observed with the addition of this prebiotic are beneficial to cats, however, further studies are needed to ascertain whether the inclusion of a higher dose of this prebiotic can provide more pronounced effects on felines.

## Methods

All procedures were approved by the Ethics Committee in the Use of Animals of the School of Veterinary Medicine and Animal Science of University of São Paulo, protocol number: 3283091014/2016.

### Experimental diets

The diets followed the AAFCO [40] recommendation for adult cat maintenance. A control diet was formulated and used as a basis for two other diets: YAM 0.3 (the control diet supplemented with 0.3% YAM) and YAM 0.6 (the control diet supplemented with 0.6% YAM) (Table 6). The animals were feeding three times a day with one of the three experimental extruded diets.

**Table 6:** Ingredients and chemical composition of experimental diets

	Diets^a^		
Item	CD	YAM 0.3	YAM 0.6
Ingredients, g/kg dry matter			
Corn starch	10.0	7.0	4.0
Original XPC^¶^	–	3.0	6.0
Corn	453.5	453.5	453.5
Poultry by-product meal	300.8	300.8	300.8
Corn gluten 60%	93.9	93.9	93.9
Poultry fat	92.1	92.1	92.1
Liquid flavor enhancer	20.0	20.0	20.0
Powder flavor enhancer	5.0	5.0	5.0
Potassium chloride	4.3	4.3	4.3
Salt	3.5	3.5	3.5
Calcium carbonate	4.0	4.0	4.0
Vitamins and mineral premix^b^	5.0	5.0	5.0
Choline chloride 60%	4.0	4.0	4.0
Mold inhibitor	2.0	2.0	2.0
Antioxidant^§^	0.4	0.4	0.4
Taurine	1.5	1.5	1.5
Chemical composition, g/kg dry matter			
Dry matter, g/kg as fed	942.1	914.6	912.7
Crude protein	345.0	328.0	332.4
Acid-hydrolyzed fat	151.8	174.6	16.05
Crude fiber	20.2	24.1	25.3
Ash	65.4	75.3	72.1
Nitrogen-free extract	417.7	398.0	409.7
Calcium	11.3	13.9	13.0
Phosphorus	11.3	11.2	10.8
Gross energy, kcal/kg	4744	4567	4513

^a^CD (control diet); YAM 0.3 (control diet containing 0.3% yeast with active metabolites); YAM 0.6 (control diet containing 0.6% of yeasts with active metabolites)

Original XPC composition: crude protein - 15%; crude fat - 1.5%; crude fiber – 25%; ash – 9%; moisture 11%; arginine – 0.73%; cystine - 0.33%; glycine – 0.93%. histidine - 0.44%; isoleucine – 0.57%; leucine – 1.03%; lysine (total) – 0.87%; methionine – 0.28%; phenylalanine – 0.61%; proline – 0.93%; threonine – 0.60%; tyrosine 0.55%; tryptophan - 0.19%; valine – 0.78%; starch – < 10%; ADF – 24.68%; NDF - 37.74; calcium – 0.56%; chloride – 0.37%; magnesium – 0.39%; phosphorus – 0.54%; potassium – 2.57%; sodium - 0.09%; sulphur – 0.46%.

^b^Supplements per kilogram of product: iron 100 mg; copper 10 mg; manganese 10 mg; zinc 150 mg; iodine 2 mg; selenium 0.3 mg; vitamin A 18000 IU; vitamin D 1200 IU; vitamin E 200 IU; thiamine 6 mg; riboflavin 10 mg; pantothenic acid 40 mg; niacin 60 mg; pyridoxine 6 mg; folic acid 0.30 mg; vitamin B_12_ 0.1 mg. Antioxidants: BHA and BHT

The amount of food offered to the cats was calculated by the National Research Council [18] equation for adult cats, as follows: 100 x (BW)^0.67^ = kcal/day. Regarding feed intake, all animals have ingested the proposed amount to maintain BW.

### Animals and experimental design

The study was conducted at Premier Pet Nutritional Development Center, Dourado, Brazil, along with the Department of Animal Nutrition and Production at School of Veterinary Medicine and Animal Science, University of São Paulo, Pirassununga, Brazil.

Thirty healthy adult domestic cats of mixed sexes, ten per group, with a mean initial age of 9.44 ± 5.35 years and a mean initial BW of 4.19 ± 0.83 kg, were distributed in two unbalanced randomized blocks according to age (adult and senior). They were divided in blocks to eliminate the age influence over the evaluated parameters. Each group was composed of three animals younger than 5 years old and the remaining were older than 10 years old.

The study period consisted of 37 days, divided into four phases: I) food adaptation (days 1 to 21); II) fecal collection for digestibility and fecal score (days 22 to 29); III) fecal collection for microbiota and fermentation products (days 30 to 34); and IV) blood collection for immunological parameters (days 35 to 37).

Cats were individually housed in clean metabolic cages (56 × 58 × 115 cm) in a temperature-controlled room at the laboratory at Premier Pet for the collection of feces. The BW of the animals was controlled weekly.

### Digestibility and fecal score

Digestibility was measured by total collection of feces without urine collection. The amount of food intake was recorded daily. The feces were stored in a freezer (− 15 °C) and, at the end of the study period, were analyzed at the Multiuser Laboratory of Animal Nutrition and Bromatology of the Department of Animal Nutrition and Production at School of Veterinary Medicine and Animal Science, University of São Paulo, Pirassununga, Brazil.

Product samples were dehydrated in a forced ventilation chamber at 55 °C for 72 h, according to the method proposed by AOAC (2006) (method 930.15). The diets were ground in a grain mill with a 1 mm sieve, and DM was determined by oven drying at 105 °C. Nitrogen content was determined by the Kjeldahl method, and it was used to calculate CP. CF content was determined by the Weende method. AHF was determined by the traditional method, as specified by AOAC [41] (method 954.02), using a Soxhlet system with previous acid hydrolysis. Ash content was determined using the method proposed by AOAC [41] (method 942.05). The NFE were calculated using the formula: NFE = 100 - (%CP + %AHF + %CF + %ash). The OM was calculated using the formula: MO = 100 - %ash.

The GE content of the diets, feces, and urine was determined in a bomb calorimeter (1281, PAAR Instrument Company, Illinois, USA) at the Animal Nutrition Laboratory of São Paulo State University, Jaboticabal, Brazil. All samples were analyzed in duplicate, and the analysis was repeated when the variation was more than 5%.

During the fecal collection, fecal quality was scored on the following scale: 1 = watery: liquid that can be poured; 2 = soft, unformed: stool assumes shape of container; 3 = soft, formed, moist: softer stool that retains shape; 4 = hard, formed, dry stool: remains firm and soft; 5 = hard, dry pellets: small, hard mass [42].

### Fecal pH, lactate, ammonia and fermentation products

Feces were collected for pH and fermentation products up to 30 min after defecation. Fecal pH was measured by direct insertion of the probe of a pH meter (Digimed, DM-20, São Paulo, Brazil) into a solution of homogenized feces. A 9:1 solution of distilled water and feces was used, according to an adapted version of the method described by Walter et al. [43].

Lactic acid was measured by Pryce’s colorimetric method [44] (Quick-Lab Spectrophotometer, Drake, São José do Rio Preto, Brazil) at the Multiuser Laboratory of Animal Nutrition and Bromatology of the Department of Animal Nutrition and Production at School of Veterinary Medicine and Animal Science, University of São Paulo, Pirassununga, Brazil.

Fecal ammonia was measured according to the method of Urrego et al. [45] using a Kjeldahl nitrogen system (TE Tecnal – 036/1, Piracicaba, Brazil).

The concentrations of SCFA and BCFA were analyzed by gas chromatography (model 9001, Finnigan, San Jose, USA) according to Erwin et al. [46] with a flame ionization detector (at a working temperature of 250 °C) controlled by the software GC solution from Shimadzu. The gas chromatograph was equipped with a capillary column (Stabilwax, Restek, Bellefonte, EUA) kept at 145 °C, and helium gas served as the carrier. For calibration, we used an external standard solution with acetic, propionic, butyric, valeric, isovaleric and isobutyric acids. The total concentration of SCFA value was obtained by the sum of concentrations of the acetic, propionic and butyric acids. The total concentration of BCFA value was obtained by the sum of concentrations of the valeric, isovaleric and isobutyric acids.

### Biogenic amines

This analysis was performed in the Laboratory of Food Biochemistry in the Food Department at the Faculty of Pharmacy of University of Minas Gerais, Belo Horizonte, Brazil. Briefly, after feces collection, 0.5 g of fresh feces was collected and preserved chilled in trichloroacetic acid (5 g/100 mL). The samples were centrifuged 3 times at 10,000×g for 20 min at 4 °C, after which the supernatant was removed and analyzed by high-performance liquid chromatography [47].

### Fecal microbiota

Quantification of fecal microbiota was performed in the Laboratory of Functional Genomics in the Department of Animal Nutrition and Production at School of Veterinary Medicine and Animal Science, University of São Paulo, Pirassununga, Brazil.

. The detection of *Lactobacillus spp.* [48], *Bifidobacterium spp.* [49], *Clostridium perfringens* [50] and *E. coli*, *Hafnia alvei* and *Shigella spp.* [51] were determined by real-time PCR. For the DNA extraction, a commercial kit was utilized (QIAamp).

Centrifugation cycles were analyzed in NetPrimer to check the optimal conditions for amplification, and specificity was checked by Basic Local Alignment Search Tool (BLAST) software (National Center for Biotechnology Information – NCBI), with the amplification performed in a Rotor Gene 6000 thermocycler (Corbett Life Science, Hilden, Düsseldorf, Germany).

Amplification reactions were performed in duplicate, and the final volume of 20 μL consisted of 10 μL of Master Mix SYBR Green (Promega, São Paulo, Brazil), 30.45 μL of forward primer (100 μM), 0.45 μL of reverse primer (100 μM), and 2 nL of DNA. Water was used as a negative control in each amplification reaction. The thermocycler was programmed to hold at 95 °C for 10 min for all amplification reactions.

### Immunological tests

All immunological tests (phagocytosis test and oxidative burst in neutrophils, lymphocyte proliferation assay and immunophenotypic) were performed in the Applied Pharmacology and Toxicology Laboratory of the Pathology Department in the School of Veterinary Medicine and Animal Science of the University of Sao Paulo.

### Phagocytosis test and the oxidative burst test

Blood leukocytes (lymphocytes, neutrophils, and monocytes) were incubated with a fluorescent reagent indicating the production of reactive oxygen species; this reagent was applied in the basal state and after phagocytosis of *Streptococcus aureus* bacteria to indicate the percentage and intensity of phagocytosis. Cells were incubated with the reagent dichlorofluorescein diacetate (DCFH-DA) in phosphate-buffered saline (PBS) and maintained at 37 °C for 20 min. DCFH-DA is a non-fluorescent cell-permeable dye that becomes fluorescent upon oxidation [52]. After the incubation period, the red blood cells were disrupted with a lysis solution, and the samples were washed with PBS until they appeared clear. The samples were then read on a FACSCalibur.

### Blood lymphocyte collection

The total peripheral blood was diluted in PBS at a 1:1 ratio, carefully transferred to Ficoll solution with a density of 1077 g/dL (GE Healthcare, Amersham, USA) at a 2:1 ratio and centrifuged continuously at 400×g for 20 min at 23 °C to separate the mononuclear cells. After centrifugation, the mononuclear cells were collected from the interface (between the plasma and the Ficoll) and washed twice by dilution in 10 mL of PBS and centrifugation at 300×g for 5 min at 4 °C. The leukocyte pellet was resuspended in 1 mL of RPMI medium (Gibco), and a 10 μL aliquot was withdrawn to count viable cells. Ten microliters of Trypan blue were added to this aliquot, and the cells were counted in a Neubauer chamber to allow adjustment of the number of cells in each assay.

### Lymphocyte proliferation assay

The blood lymphocytes were labeled with carboxyfluorescein diacetate succinimidyl ester (CFSE) and seeded in triplicate at a concentration of 5 × 10^4^ cells/well in 96-well U-bottom plates with or without ConA (5 μg/mL) and PHA (10 μg/mL), which are substances with mitogenic potential in T lymphocytes. The plates were incubated in a 5% CO_2_ incubator at 40 °C for 48 h. CFSE binds covalently to intracellular molecules through carboxyfluorescein, a fluorescent dye. Therefore, when a CFSE labeled cell divides, each of its daughter cells carries half the number of molecules labeled with carboxyfluorescein; thus, the number of cell divisions can be evaluated by measuring the corresponding decrease in cell fluorescence by flow cytometry. The entry of the dye into the cell is possible because it is in its diacetylated form [carboxyfluorescein diacetate (CFDA), SE]. The acetate makes the dye highly membrane permeant, allowing its rapid flow into the cell. The esterases present in the cell cleave CFDA acetates, yielding CFSE, which is much less membrane permeant and consequently becomes concentrated in the cellular interior (Quah and Parish, 2010). At the end of three days, the cells were examined by flow cytometry (FACSCalibur) and analyzed by FlowJo.

### Immunophenotypic quantification

Flow cytometric analysis was used to perform immunophenotyping to quantify T helper cells (CD4+), cytotoxic T cells (CD8+), and the CD4/CD8 ratio. Mononuclear cells (2 × 105 cells/mL) were incubated in microtubes (1.5 mL) with anti-CD4 (1:10) and anti-CD8 [(1:20) antibodies (Serotec, BioLegend, San Diego, USA), diluted in 100 μL of cytometry buffer (PBS containing 0.5% bovine serum albumin and 0.02% sodium azide)], and then washed with buffer. We used gating criteria to identify a population of lymphocytes with low size and low complexity. Immunolabelling was performed according to the method of Gil et al. [53]. We analyzed 10.000 cells by flow cytometry.

### Statistical analysis

Data were analyzed by the statistical software SAS (version 9.2, SAS Institute Inc., Cary, USA); the normality of errors was verified by the Shapiro-Wilk test (PROC UNIVARIATE), after which the variances were compared by the F-test. Variables that did not meet the assumptions were transformed with a log or square root transformation and were analyzed by PROC GLM in SAS by simple polynomial regression. To determine differences between treatments, we applied the method of least-square means using Tukey’s test. Values of *p* < 0.05 were considered significant.

## Data Availability

All data generated or analyzed during this study are included in this published article.

## Abbreviations

YAM: Yeast with active metabolites
CD: Control diet
YAM 0.3: Control diet with 0.3% yeast with active metabolites
YAM 0.6: Control diet with 0.6% yeast with active metabolites
BA: Biogenic amines
SCFA: Short-chain fatty acids
BCFA: Branched-chain fatty acids
MOS: Mannan oligosaccharides
BW: Body weight
DM: Dry matter
OM: Organic matter
CP: Crude protein
AHF: Acid-hydrolyzed fat
NFE: Nitrogen-free extract
CF: Crude fiber
GE: Gross energy
ConA: Concanavalin A
PHA: Phytohemagglutinin
YCW: Yeast cell wall
FOS: Fructooligosaccharides
GOS: Galactooligosaccharides
DCFH-DA: Reagent dichlorofluorescein diacetate
CFSE: Carboxyfluorescein diacetate succinimidyl ester
CFDA: Carboxyfluorescein diacetate
